# Loss of heterozygosity as a marker of homologous repair deficiency in multiple myeloma: a role for PARP inhibition?

**DOI:** 10.1038/s41375-018-0017-0

**Published:** 2018-02-02

**Authors:** Charlotte Pawlyn, Andrea Loehr, Cody Ashby, Ruslana Tytarenko, Shayu Deshpande, James Sun, Kyle Fedorchak, Tariq Mughal, Faith E. Davies, Brian A. Walker, Gareth J. Morgan

**Affiliations:** 1 The Institute of Cancer Research, London, UK; 2 Clovis Oncology Inc., San Francisco, CA USA; 3 Myeloma Institute, University of Arkansas for Medical Sciences, Little Rock, AR USA; 4 Foundation Medicine, Cambridge, MA USA

## Abstract

PARP inhibitors can induce synthetic lethality in tumors characterized by homologous recombination deficiency (HRD), which can be detected by evaluating genome-wide loss of heterozygosity (LOH). Multiple myeloma (MM) is a genetically unstable tumor and we hypothesized that HRD-related LOH (HRD-LOH) could be detected in patient samples, supporting a potential role for PARP inhibition in MM. Using results from targeted next-generation sequencing studies (FoundationOne^®^ Heme), we analyzed HRD-LOH in patients at all disease stages (MGUS (*n* = 7), smoldering MM (SMM, *n* = 30), newly diagnosed MM (NDMM, *n* = 71), treated MM (TRMM, *n* = 64), and relapsed MM (RLMM, *n* = 234)) using an algorithm to identify HRD-LOH segments. We demonstrated HRD-LOH in MM samples, increasing as disease progresses. The extent of genomic HRD-LOH correlated with high-risk disease markers. Outcome of RLMM patients, the biggest clinical group, was analyzed and patients with HRD-LOH above the third quartile (≥5% HRD-LOH) had significantly worse progression-free and overall survival than those with lower levels (*p* < 0.001). Mutations in key homologous recombination genes account for some, but not all, of the cases with an excess of HRD-LOH. These data support the further evaluation of PARP inhibitors in MM patients, particularly in the relapsed setting with a high unmet need for new treatments.

## Introduction

Multiple myeloma (MM) is malignancy of plasma cells that has been extensively characterized at a molecular level over recent years. It can be split into groups of hyperdiploid and non-hyperdiploid cases, the latter being defined by a number of recurrent chromosomal translocations. More recently the mutational patterns underlying MM have been described, with those affecting *KRAS* and *NRAS* being the most common and identified in nearly 50% of all cases [1–3]. Targeting specific mutations has therapeutic potential but is likely to be limited by the intra-clonal and spatial genetic heterogeneity typical of MM and the ability of the tumor cells to signal via alternate pathways [4–7]. A different strategy is to target the common downstream consequences of tumor acquired mutations. In this respect, one approach would be to target homologous recombination deficiency (HRD) resulting from mutation of DNA repair pathway genes or other mechanisms. This could be achieved utilizing a synthetic lethal approach with PARP inhibition, as has been demonstrated in other tumor types.

Homologous recombination repair (HRR) is an important mechanism that maintains DNA integrity especially after double-strand DNA breaks. The biochemical mechanisms that underlie HRR have been well described with double-strand breaks being detected by the MRE11-RAD50-NBS1 (MRN) complex, which then binds DNA and recruits ATM and BRCA1. BRCA2 and its partner PALB2 localize to exposed single-stranded DNA allowing DNA polymerase to use the homologous DNA as a template for DNA repair. When HRR is impaired (HRD), often as a result of genetic changes in the key players, less-precise forms of DNA repair are used such as non-homologous end joining. This results in the induction of point mutations or frequent deletions and has been implicated in the progression of a range of cancers [8, 9]. These areas of chromosomal deletion can be identified and quantified by determining the extent of loss of heterozygosity (LOH) across the genome.

Acquired mutations in *BRCA1*and *BRCA2* result in HRD and is a key mechanism by which a number of different cancers progress [9]. Mutations in this limited set of genes cannot, however, fully explain the extent of clinically relevant HRD and a range of additional mutations have been identified at other points in the pathway as well as epigenetic changes that may be responsible for abnormalities that would not be detectable by mutation analysis [9–11]. The extent of genome-wide LOH provides a single global assessment of HRD irrespective of causative lesion, and the potential for using it as a therapeutic target [12].

Identifying HRD is therapeutically relevant because PARP inhibitors can induce synthetic lethality in tumors with HRD and two such drugs are currently approved by the FDA for the treatment of *BRCA*-mutated ovarian cancer, rucaparib, and olaparib [13, 14], while niraparib is approved for maintenance treatment in ovarian, fallopian tube, or peritoneal cancer patients. PARP is required to repair single-strand breaks by base excision repair, so when it is inhibited these single-strand breaks lead to stalled replication forks and double-strand breaks. In cells with functioning HRR, these can be repaired but in cells with HRD they cannot, leading to a level of genomic instability that overwhelms the cell resulting in apoptosis. A key example of this therapeutic approach is provided by ovarian cancer where patients in the ARIEL2 study with a *BRCA* mutation or with a “BRCA-like” LOH-based signature but no *BRCA* mutation had better responses and longer progression-free survival to the PARP inhibitor rucaparib than *BRCA* wild-type patients lacking this signature [12].

Large areas of chromosomal loss that have been previously identified in myeloma, e.g. del[1p], del[13q] are well recognized and have been associated with adverse outcomes [15, 16]. Smaller regions of loss across the genome that may be related to HRD are less well characterized. Mutations in some DNA damage response genes have been identified but the wider extent of the LOH phenotype is unknown. In this study, we have characterized and quantified the presence of global HRD-related LOH (HRD-LOH) in MM patients and have used it as an indicator of HRD that could provide a therapeutic rational for PARP inhibition in a subgroup of cases.

## Methods

We analyzed 406 myeloma cases treated at the University of Arkansas for Medical Sciences (UAMS) at all stages of disease: monoclonal gammopathy of undetermined significance (MGUS, *n* = 7), smoldering myeloma (*n* = 30), newly diagnosed myeloma (*n* = 71), treated myeloma (*n* = 64), and relapsed myeloma (RLMM, *n* = 234). CD138-positive plasma cells were selected from patient samples and DNA extracted. DNA underwent targeted next-generation sequencing (FoundationOne^®^ Heme) interrogating 405 cancer-related genes and 3543 single-nucleotide polymorphisms (SNPs) across the genome as previously described [17, 18]. Targeted sequencing was performed as part of patient disease work-up and review of the data was approved by the UAMS institutional review board.

To identify genomic LOH, an algorithm was developed to quantify the percentage of the genome interrogated with LOH. Briefly, minor-allele frequencies of SNPs and the copy-number profile of the 22 autosomal chromosomes were used to identify LOH. We excluded from this percentage events that are unlikely to be caused by HRD mechanisms but are common in myeloma, such as whole-chromosome or chromosome-arm loss. The percentage of genomic HRD-LOH for each sample was calculated as the sum of the lengths of the HRD-LOH segments divided by the length of the interrogated genome. An in-depth description of the algorithm can be found in Swisher et al. [12], supplemental information.

Percentage HRD-LOH was examined by the presence or absence of a deleterious mutation in 1 of 23 homologous recombination related genes that had been interrogated by NGS: *ATM*, *ATR*, *ATRX*, *BARD1*, *BAP1*, *BLM*, *BRCA1*, *BRCA2*, *BRIP1*, *CDK12*, *CHEK1*, *CHEK2*, *FANCA*, *FANCC*, *FANCD2*, *FANCE*, *FANCF*, *FANCG*, *FANCL*, *MRE11A*, *PALB2*, *RAD50*, *RAD51*. Matched gene expression profiling (GEP) was performed using Affymetrix U133 Plus 2.0 Arrays and GEP70-defined risk status, proliferation index (PI), and molecular subgroups were calculated, as previously described [19, 20].

## Results

**Fig. 1:**
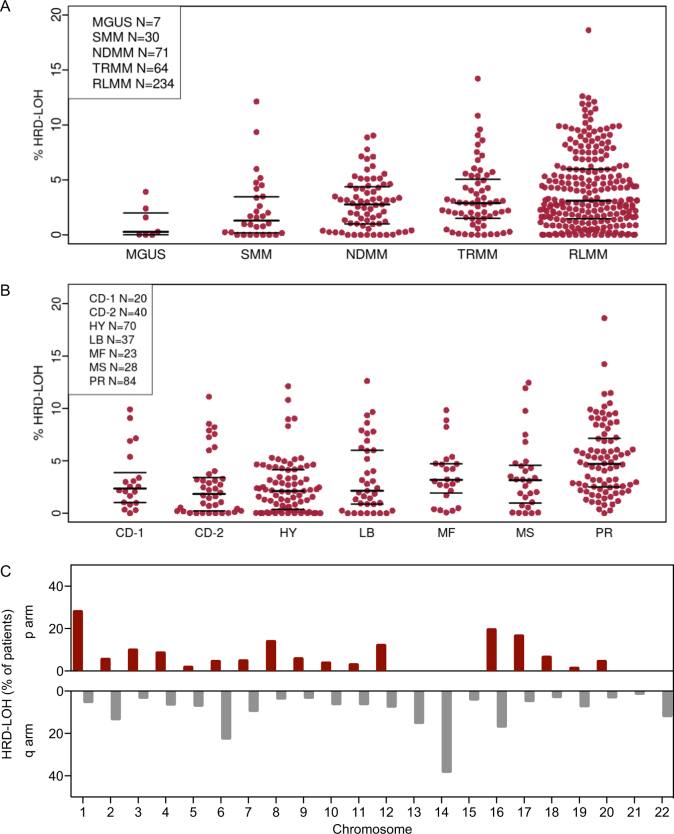
**a** Percentage of genomic HRD-LOH by disease stage. **b** Percentage of genomic HRD-LOH by UAMS molecular subgroup. The raw data points and the median and inter-quartile range for each data set are shown. **c** Distribution of HRD-LOH shown as the percentage of patients with HRD-LOH >1% at each genomic locus across the autosomal chromosomes. For the acrocentric chromosomes, 13, 14, 15, 21, and 22, only the *q* arm was considered as estimates of HRD-LOH in the *p* arm would be unreliable

The median level of genomic HRD-LOH was 2.8% across all samples with a subset of 25% of patients having levels of >5% and around 4% of patients having high levels at >10%. There was no difference in HRD-LOH between genders. The level of HRD-LOH increased significantly with increasing disease stage from a median of 0.3% in MGUS to 3.1% in RLMM, (analysis of variance; *F* = 4.7, *p* = 0.0011), Fig. 1a, suggesting that HRD-LOH may be acquired throughout disease evolution as a result of the acquisition of additional genetic events.

To examine the association of molecular subgroup with the extent of HRD-LOH, we determined the distribution of HRD-LOH across the UAMS molecular subgroups [20]. We found that HRD-LOH was highest in the proliferation (PR), MAF (MF), and MMSET/FGFR3 (MS) subgroups, Fig. 1b. It is notable that higher levels of HRD-LOH were seen in these subgroups associated with an increased incidence of high-risk disease. Consistent with this observation, the extent of HRD*-*LOH correlated significantly with GEP70-defined risk status [21], (*R* = 0.42, *p* = 7 × 10^−15^). There was also a significant correlation with the gene expression defined proliferation index (PI) [22], another marker of high-risk disease (*R* = 0.39, *p* = 2 × 10^−12^). HRD-LOH was distributed as shown in Fig. 1c. The pattern of distribution was not dominated by specific loci with high rates of HRD-LOH, which was spread throughout the genome.

**Fig. 2:**
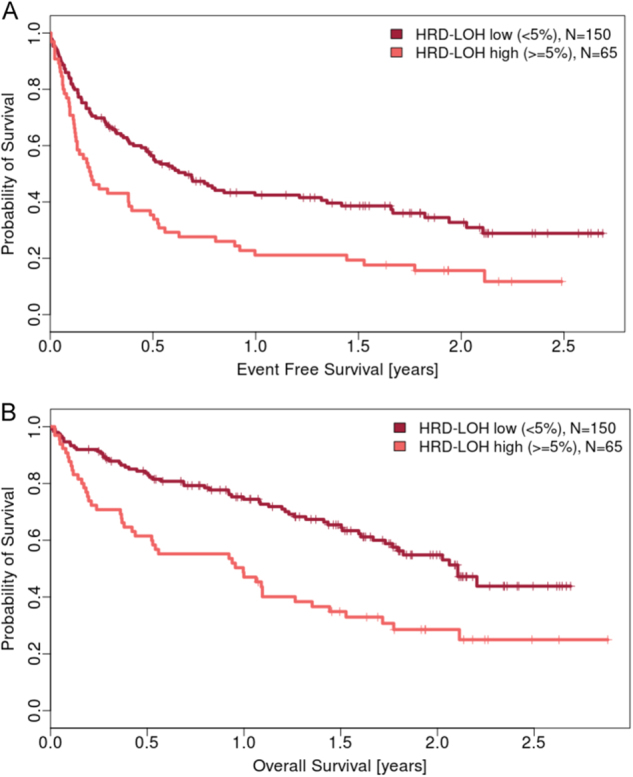
**a** Event-free survival by genomic HRD-LOH. Kaplan–Meier plots demonstrating the probability of survival for those patients with genomic HRD-LOH <5% (*n* = 150, median 0.512 years) vs. ≥5% (*n* = 65, median 0.197 years). Hazard ratio 1.79 (1.3, 2.5); *p* = 0.00065. **b** Overall survival by genomic HRD-LOH. Kaplan–Meier plots demonstrating the probability of survival for those patients with genomic HRD-LOH <5% (*n* = 150, median 1.266 years) vs. ≥5% (*n* = 65, median 0.939 years). Hazard ratio 2.18 (1.5, 3.2); *p* = 8.23e−05

We went on to examine the association of HRD-LOH with PFS and OS in the largest group of patients, those with RLMM. We found that patients with levels of HRD-LOH in the top quartile for the whole population (≥5%) had a significantly shorter event-free survival (EFS) than those with lower levels. Patients with HRD-LOH <5% (*n* = 150), had a median EFS of 0.51 years, while those with HRD-LOH ≥5% (*n* = 65) had a median EFS of 0.20 years (HR 1.79, *p* = 6.5 × 10^−4^). This signal was stable across cutoff values from 1 to 9% with the optimal cut point defined as 5%, Fig. 2a. A similar pattern was seen for overall survival (OS). Patients with HRD-LOH <5% (*n* = 150), had a median OS of 1.3 years, while those with HRD-LOH ≥5% (*n* = 65 had a median OS of 0.94 years (HR 2.18, *p* = 8.2 × 10^−5^)). There was again a significant difference seen at all cutoff values 1–9% and an optimal cutoff of 5%, Fig. 2b. In a multivariate Cox regression analysis, we find that HRD-LOH as a biomarker of poor prognosis overlaps in large parts with other prognostic factors, such as the PR subgroup, GEP70, and PI. In particular, GEP70 captures the patient population with poor outcome better than a combination of HRD-LOH and GEP70, as indicated by a non-significant *p* value for HRD-LOH in the multivariate model for both OS and EFS. In combination with PR and PI, LOH makes a small but significant contribution to enhancing the biomarkers of poor prognosis PR and PI on their own (Table 1).

**Table 1:** Multivariate Cox regression analysis of event-free and overall survival

Event-free survival	Wald interaction *p* value	*p* value of HRD-LOH contribution
HRD-LOH*PR	<0.0001	0.01
HRD-LOH*GEP70	<0.0001	0.08
HRD-LOH*PI	<0.0001	0.02
*Overall survival*	*Wald interaction p value*	*p value of HRD-LOH contribution*
HRD-LOH*PR	<0.0001	0.002
HRD-LOH*GEP70	<0.0001	0.39
HRD-LOH*PI	<0.0001	0.04

**Fig. 3:**
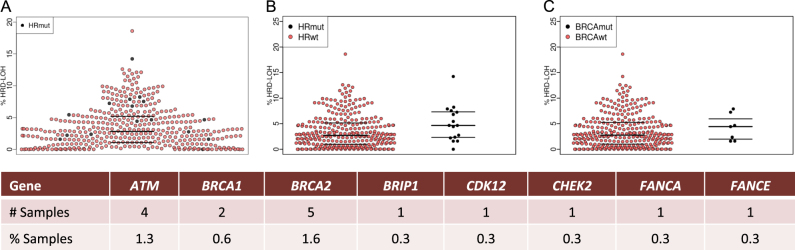
**a** Genomic HRD-LOH across all myeloma samples with those carrying a mutation in a gene associated with homologous recombination indicated in black (HRmut). **b** Genomic HRD-LOH by presence of HRmut or wild type (HRwt). Wilcoxon test for difference between groups, *p* = 0.056. **c** Genomic HRD-LOH by presence of a *BRCA1/2* mutation. Wilcoxon test for difference between groups, *p* = 0.383. The raw data points and the median and inter-quartile range for each data set are shown. The number and percentage of patients with a mutation identified in each of the genes indicated is shown in the table below the figure

We next analyzed paired mutation data, which was available for 311/406 cases. Deleterious mutations in any of the 23 genes sequenced that have been associated with homologous recombination (*ATM*, *ATR*, *ATRX*, *BARD1*, *BAP1*, *BLM*, *BRCA1*, *BRCA2*, *BRIP1*, *CDK12*, *CHEK1*, *CHEK2*, *FANCA*, *FANCC*, *FANCD2*, *FANCE*, *FANCF*, *FANCG*, *FANCL*, *MRE11A*, *PALB2*, *RAD50*, and* RAD51*) were identified in 5.1% (16/311) of patients, Fig. 3a. *ATM* (4/311, 1.3%) and *BRCA2* (5/311, 1.6%) were the most frequently mutated genes. The sample with the second highest HRD-LOH of 14.2% had a deleterious *ATM* mutation. Samples with deleterious mutations in any homologous recombination gene had a higher median genomic HRD-LOH than those without (4.66 vs. 2.85%, *p* = 0.056), Fig. 3b. This comparison met statistical significance when limited to mutations found in genes most highly associated with homologous recombination (*ATM*, *BARD1*, *BRCA1*, *BRCA2*, *BRIP1*, *CDK12*, *CHEK2*, *FANCA*, *PALB2*, *RAD51*, *p* = 0.049). The difference was also apparent when *BRCA1/2* deleterious mutations were examined alone Fig. 3c. As can be seen in Fig. 3a mutations in HR genes could account for some, but not all, of the top quartile of HRD-LOH cases suggesting there are other mechanisms mediating this phenotype.

## Conclusions

We show, for the first time in myeloma, that it is possible to quantitate the extent of genome-wide HRD-LOH and that there is a group of patients with an excess of such change that is associated with impaired outcomes. Genome-wide HRD-LOH increases as the disease progresses and also within higher risk groups. The median level of HRD-LOH across the population as a whole is relatively low at 2.8% compared to ovarian cancer (~16%), but is still a significant pathologic mechanism in the cases with high levels and poor outcomes.

We show that mutations in key homologous recombination genes account for some but not all of the cases with an excess of HRD-LOH suggesting there are other mechanisms that are also responsible for the phenotype. These may include epigenetic phenomena such as homologous recombination gene silencing by DNA methylation as seen in breast and ovarian cancer [23], but these mechanisms were not analyzed in this study.

The group of patients identified with an excess of HRD-LOH may have associated features of 'BRCAness' and so may be amenable to PARP inhibition. The concept of BRCAness has been developed in ovarian cancer where cases share features with those with mutational inactivation of *BRCA* genes, such as  sensitivity to platinum-based therapies and PARP inhibitors. In ovarian cancer, the optimal cutpoint for percentage of genome-wide HRD-LOH that predicted response to PARP inhibitor therapy was ascertained to be 14% within the ARIEL2 trial [12]. While we have shown the distribution and extent of HRD-LOH in myeloma, as well as the optimum cutpoint associated with adverse outcomes, the relevant cutoff to predict response to PARP inhibition in this disease will need to be further explored in clinical studies.

A number of lines of pre-clinical evidence support a clinical exploration of PARP inhibitors in myeloma. *In vitro* responses in myeloma cell lines and primary patient samples have been studied using the PARP1/2 inhibitor ABT-888. Despite demonstration of induction of DNA damage, the use of ABT-888 as a single agent was not effective, but it is unknown whether these cells had either mutations in DNA repair genes or had an excess of HRD-LOH [24, 25]. Sensitivity to PARP inhibition was induced, either by chemically inhibiting the DNA damage response pathway [25] or by inducing increased BRCAness with the proteasome inhibitor bortezomib [24]. In both studies, this synthetic lethal effect was associated with a downregulation of HRR genes. Identifying patients with inherent BRCAness who may be more likely to respond to single agent PARP inhibition may prove fruitful. In addition, expanding such studies to utilize combinations with both cisplatin and proteasome inhibition would be clinically very useful because this type of genomic instability is more prevalent in high-risk disease states where therapeutic options are limited.
